# MEDICARE ENROLLEES WITH COMPLEX HEALTH NEEDS FROM PUERTO RICO

**DOI:** 10.1093/geroni/igae098.0195

**Published:** 2024-12-31

**Authors:** Maricruz Rivera-Hernandez, Matthew Campos

**Affiliations:** Brown University School of Public Health, Providence, Rhode Island, United States; Brown University School of Public Health, Providence, Rhode Island, United States

## Abstract

Older adults in Puerto Rico have higher rates of chronic conditions and poverty rates compared to adults in the US mainland (Matos-Moreno et al., 2022; U.S. Census Bureau, 2019). In addition, the island of Puerto Rico has faced unique challenges related to infrastructure damages as a result of Hurricane Maria in 2017 (Florido, 2020), and further continues to face disparities in quality of care for older adult populations (Rivera-Hernandez et al., 2021). While the proportion of older adults with health insurance in Puerto Rico is comparable to older adults in the U.S. mainland, disparities in quality and access to care exist for older adults in Puerto Rico (Matos-Moreno et al., 2022). Thus, the purpose of this study was to characterize older adults with complex care needs enrolled in Medicare Advantage (MA) from Puerto Rico. We used data from the Medicare Health Outcomes Survey and the Master Beneficiary Summary File from 2010 to 2019. We classified enrollees into high-need and non-high-need adults (DuGoff et al., 2019). We identified about 46,699 beneficiaries from Puerto Rico. Approximately, 57.09% were identified as high-need adults. A higher proportion of high-need older adults were female (56.59% vs. 50.99%), married (50.95% vs. 58.68%), had high school education (28.09% vs. 22.88) and household income of less than $10,000 (35.48% vs. 28.83%) compared to non-high-need beneficiaries. With physicians and health care providers leaving Puerto Rico, older adults with high-needs may be experiencing health care challenges. These issues may be contributing to the exodus of older adults from Puerto Rico.

